# *CSN1S1* and *CSN1S2*: Two Remarkable Examples of Genetically Modulated Alternative Splicing via Identification of Allele-Specific Splicing Events

**DOI:** 10.3390/genes16091011

**Published:** 2025-08-27

**Authors:** Gianfranco Cosenza, Andrea Fulgione, Emanuele D’Anza, Sara Albarella, Francesca Ciotola, Alfredo Pauciullo

**Affiliations:** 1 Department of Agricultural Science, University of Naples Federico II, Piazza Carlo Di Borbone 1, 80055 Portici, Italy; gianfranco.cosenza@unina.it (G.C.); andrea.fulgione@unina.it (A.F.); 2 Department of Veterinary Medicine and Animal Production, University of Naples Federico II, Via Delpino 1, 80137 Naples, Italy; emanuele.danza@unina.it (E.D.); francesca.ciotola@unina.it (F.C.); 3 Department of Agricultural, Forest and Food Sciences, University of Turin, 10095 Grugliasco, Italy; alfredo.pauciullo@unito.it

**Keywords:** mRNA, dairy livestock species, caseins, mutations, splice sites

## Abstract

Splicing regulatory sequences are cornerstones for exon recognition. Mutations that modify them can severely compromise mRNA maturation and protein production. A wide range of mutations, including SNPs and InDels, can influence splicing regulatory signals either directly (e.g., altering canonical donor and acceptor dinucleotides) or indirectly (e.g., creating cryptic splice sites). *CSN1S1* and *CSN1S2* genes encode for the two main milk proteins, αs1 and αs2 caseins, respectively. They represent a remarkable and unique example of the possibilities for alternative splicing of individual genes, both due to the high number of alternative splices identified to date and for recognized allele-specific splicing events. To date, at least 13 alleles of *CSN1S1* originating from mutations that affect canonical splice sites have been described in *Bos taurus* (*CSN1S1* A, A1, and H), *Ovis aries* (E, H, and I), *Capra hircus* (D and G), *Bubalus bubalis* (E, F) and *Camelidae* (A, C, and D). Similarly, allele-specific splicing events have been described at the *CSN1S2 locus* in *B. taurus.* (*CSN1S2* D), *C. hircus* (*CSN1S2* D), *B. bubalis* (*CSN1S2* B, B1, and B2), *Equus asinus* (*CSN1S2* I B), and *Camelidae*. This review highlights that mutations affecting canonical splice sites, particularly donor sites, are significant sources of genetic variation impacting the casein production of the main dairy livestock species. Currently, a key limitation on this topic is the lack of detailed functional and proteomic studies. Future research should leverage advanced omics technologies like long-read transcriptomics and allele-resolved RNA sequencing to characterize these splicing mechanisms, guiding precision breeding strategies.

## 1. Introduction

Alternative splicing (or non-constitutive splicing) is an intriguing mechanism able to expand proteome complexity and gene expression. It plays a multifaceted role in several biological processes, including the modulation of gene expression [1] and the regulation of cell and tissue development and differentiation [2]. Thus, it has implications for human diseases [1], and it contributes to the evolution of complex organisms [3]. A major factor influencing the intricate mechanism of RNA maturation is the highly fragmented architecture of genes, which accounts for the frequent occurrence of alternative splicing. Exon skipping is particularly frequent when the coding region is divided into multiple short exons, as the maturation of long primary transcripts is an intricate process requiring many successive steps [4]. Genes with multiple alternative splicing forms are expected to contain more regulatory sequences that may serve as potential target for mutations. Some reported mutations disrupt splicing by either generating new splice sites or interfering with regulatory elements. Therefore, the role of splicing mutations in gene expression and regulation may be more significant than previously believed [5].

Despite their potential significance, the number of reported mutations that alter RNA splicing remains relatively small and less investigated in the literature compared to other mutations, particularly those occurring in exons that affect a protein’s functional or structural properties [6].

In this respect, very extensive non-constitutive splicing events (both alternative splicing and genetically modulated alternative splicing) were observed in the casein transcripts of various species, in particular at *CSN1S1* and *CSN1S2* loci, encoding for two main milk proteins, αs1 and αs2 caseins, respectively [4,5,6,7,8,9].

In this review, we examine sequence changes in the *CSN1S1* and *CSN1S2* genes that impact transcript structure. Although factors like spliceosomal recognition and RNA binding proteins play a role in both normal and mutation-derived alternative mRNA splicing, we specifically concentrate on genetic changes that disrupt constitutive splicing, often leading to abnormal phenotypes.

## 2. mRNA Splicing

A major process regulating gene expression in eukaryotes is precursor messenger RNA (pre-mRNA) splicing. It consists in the removal of intron sequences and the linear joints of remaining exons in the order in which they were in the originally transcribed DNA sequence to codify for a specific final protein [10].

The leading factor in this process is the spliceosome, an intricate molecular complex of proteins and RNA. It is composed of the small nuclear ribonucleic particles (sn-RNPs) U1, U2, U4/U6, and U5, along with other splicing factors (non-RNPs). It is responsible for the accurate removal of introns from pre-mRNA. This is achieved by identifying specific, conserved sequence elements at the exon–intron boundaries: the 5′ and 3′ splice sites, the branch point, and the polypyrimidine tract [11,12,13].

The donor splice site, at the 5′ end of an intron, is characterized by the dinucleotide GT immediately after the cut point where the intron is removed from the preceding exon. Conversely, the acceptor splice site, at the 3′ end of an intron, has the dinucleotide AG just before the cut point where the intron is removed from the following exon. It has been estimated that GT-AG splice site pairs represent more than 99% of all the canonical splice sites. However, several less common instances were discovered where non-canonical splice pairs were processed by related, but distinct, splicing machinery. Major non-canonical splice site combinations are GC-AG (0.56–0.69%) and AT-AC (0.05–0.09%); all sequences differing from these are non-canonical splice sites (0.02%) [14,15].

The branch point is contained within a degenerate intronic heptamer (YNYYRAY common consensus sequence in animals) called “the branch point sequence”. In eukaryotes, the lariat formation site at the branch point holds an adenosine in the sequence, while the rest of the sequence is not conserved. In most introns, this motif resides between approximately 18–40 nucleotides upstream of the 3′ splice site before a polypyrimidine tract. This region is rich in pyrimidines (C and U) of variable length, often interrupted by purines. It is a crucial cis-acting sequence element for intron removal, a process in which the pre-mRNA splicing factor U2AF65 plays a pivotal role. It directs the early steps in splice site choice by recognizing the polypyrimidine tract consensus sequence located near the 3′ splice site [16].

The type of splicing just described is defined as linear because a 5′ splice site is connected to a downstream 3′ splice site. However, it has been demonstrated that under certain conditions, the same splicing machinery and most of the splicing enhancers and silencers used for linear pre-mRNA splicing can non-colinearly splice the 5′ terminus of an upstream pre-mRNA exon with the 3′ terminus of a downstream exon.

This mechanism, named back-splicing, generates circular RNAs (circ-RNAs), a recently discovered class of single-stranded non-coding RNA (ncRNA). A prerequisite for the back-splicing mechanism is the spatial proximity of the corresponding splice sites, which is commonly achieved through the folding of the pre-mRNA into specific secondary structures. This unique splicing event results in a covalent bond between the 3′ and 5′ ends of the RNA molecule. The resulting lack of free termini renders circRNAs exceptionally stable and resistant to exoribonuclease degradation, setting them apart from their linear cognates. Back-splicing can occur in two main forms: splicing within a single exon to produce mono-exonic circRNAs, or a non-sequential joining of the 5′ terminus of an upstream exon with the 3′ terminus of a downstream exon, leading to multi-exonic circRNAs. Furthermore, similarly to protein-coding linear mRNAs, circRNAs can be subject to alternative splicing events.

Currently, it is estimated that circRNAs typically account for approximately 0.8–1% of linear messenger RNAs (mRNAs) on a global scale. Despite this prevalence, the overarching biological function of circRNAs remains poorly understood, and for years, they were largely dismissed as mere splicing artifacts. However, recent investigations have begun to uncover their diverse roles, including their ability to act as protein scaffolds, serve as microRNA (miRNA) sponges, and be translated into novel polypeptides [17,18,19].

In addition to constitutive splicing, at least two types of non-constitutive splicing have been observed: alternative splicing and allele-specific splicing.

### 2.1. Alternative Splicing

Alternative splicing is a ubiquitous regulatory mechanism and can be defined as a variation of constitutive splicing [12]. It is estimated that between 92% and 94% of all mRNAs in the human genome are alternatively spliced [20].

Alternative splicing is a cellular process in which exons from a single gene are joined in various combinations, generating multiple, related messenger RNA (mRNA) transcripts. These mRNA isoforms can be translated into a diverse set of proteins that possess distinct structures, functions, and/or subcellular localizations. Furthermore, certain splicing events can alter the mRNA reading frame, producing proteins with novel functions or leading to non-functional products. This mechanism significantly expands proteomic complexity from a limited number of genes [10].

There are several ways in which alternative splicing can be achieved: (a) skipping of one or more exons (also known as cassette exons); (b) activation of one or more cryptic splice sites and “exonization” of an intronic region following the recognition of canonical di-nucleotide splice acceptor and donor sites; (c) transition of a constitutive exon to an alternative exon; (d) alternative 5′ splice sites (alternative donors) or alternative 3′ splice sites (alternative acceptors); (e) exon shuffling, a process in which a new or a duplicated exon is inserted into a gene. The exon–intron architecture appears to be the cornerstone of alternative splicing. Long introns and short exons characterize genome architecture in vertebrates, whereas in lower eukaryotes, introns are short, and exons are long [10,15,21].

While core splice site motifs are essential, the vast majority of the regulatory information for splicing is thought to be encoded within exonic and intronic cis-regulatory elements. These elements function as binding sites for sequence-specific RNA-binding protein factor, and they in turn either promote or suppress the recognition of proximal splice sites [22].

Furthermore, the alternative inclusion of exons with weak splice sites (weak exons) in the processed mRNA is observed. The efficiency of this inclusion is contingent upon several factors, including the activity of splicing activators or repressors, the RNA secondary structure, and the promoter structure. The RNA secondary structure, alongside the primary RNA sequence, is a critical determinant of a correct splicing process. A growing body of research also demonstrates that alternative splicing occurs in both protein-coding and non-coding genes [23].

A well-known example of a post-transcriptional consequence of alternative splicing is its intricate relationship with nonsense-mediated mRNA decay (NMD). Nonsense mutations, which introduce a premature termination codon (PTC) within an exon, can trigger these regulatory pathways. The presence of a PTC has been demonstrated to induce changes in the splicing pattern of the pre-mRNA, frequently leading to the skipping of the exon containing the mutation. This splicing alteration, coupled with the direct instability caused by NMD, results in low levels of the mutant mRNA. This mechanism serves to prevent the translation of functionally defective or potentially harmful truncated proteins [24].

Alternative splicing can also serve as a quantitative regulator of gene expression. Furthermore, it may lead to the generation of transcripts that fail to produce functional proteins. These outcomes are often attributed to variations in untranslated regions (UTRs), which can modulate mRNA stability and translational efficiency, produce truncated proteins, or change the mRNA localization, thus compromising the correct function of the transcript and/or protein. So, alternative splicing represents an important mechanism of increasing proteome complexity and regulating gene expression. An evolutionary advantage of alternative splicing is its ability to enable a single gene to produce a variety of functionally distinct mRNA transcripts that are similar but not identical. This mechanism substantially increases the coding capacity and proteomic diversity of eukaryotic genomes, as it allows for the generation of multiple protein isoforms from a single genetic locus [23,25,26].

Therefore, the full characterization of transcriptome and proteome isoforms derived by alternative splicing is essential for understanding cellular differentiation and the biological diversity observed across living organisms [27].

### 2.2. Allele-Specific Splicing

Consensus splicing regulatory sequences are pivotal determinants for exon recognition, and mutations that alter these sequences can profoundly impact mRNA maturation and subsequent protein synthesis. It is well established that a wide range of genetic mutations (SNPs, InDels, etc.), in both intronic and exonic regions, can disrupt these signals. This disruption may occur either directly—by altering canonical, highly conserved, donor and acceptor dinucleotides or the branch point sequence—or indirectly, through mechanisms like the creation of cryptic splice sites.

The most frequent and impactful mutations occur at the highly conserved first two nucleotides (+1/+2) of the 5′ donor site and the last two nucleotides (–1/–2) of the 3′ acceptor site. Notably, mutations at the donor splice site are more prevalent than those at the acceptor splice site, with a reported ratio of approximately 1.5:1 [28]. The two highly conserved splice sites exhibit significantly lower variability compared to exonic sequences, with reductions of 77% and 84% for the donor and acceptor sites, respectively. Furthermore, the intronic nucleotides immediately flanking these sites—specifically, the first eight and last twelve nucleotides at the 5′ and 3′ splice sites—are also more highly conserved than the average intronic sequence [11]. When a canonical splice site is weak or a mutation activates a neighboring cryptic splice site, this alternative site can be preferentially used by the splicing machinery. The resulting outcome depends on the location of the cryptic site: if it resides within an intron, the splicing process can lead to the inclusion of an intronic fragment; conversely, if it is located within an exon, it can cause the partial or complete removal of that exon [4,14].

The allele specificity of transcript isoforms may be complete or partial. Such sequence variants often disrupt normal gene splicing, causing aberrant splicing of either a proportion or all of the transcripts produced (complete allele specificity), altering the mRNA and, consequently, the corresponding encoded protein sequences, which sometimes are aberrant or incorrectly functional. Alternatively, a single allele can produce multiple transcript isoforms (true alternative splicing). However, the relative abundance of these isoforms can exhibit significant variation between different alleles (partial allele specificity) [29,30,31].

Mutations occurring in the branch point sequence are rarer but can lead to exon skipping due to improper binding of the SF1 and U2 snRNP splicing proteins, resulting in disruption of the natural acceptor splicing site. Alternatively, they can cause intron retention, either partial or complete, if a new 3′ splice site is created [11,12,14].

Mutations affecting the branch point adenosine or its surrounding consensus sequence can disrupt the spliceosome’s ability to recognize and utilize the canonical branch point. Consequently, the splicing machinery may be prompted to utilize a nearby cryptic adenosine residue within the intron as a novel branch point. This mechanism contributes to alternative splicing events and subsequent dysregulated gene expression, which can have significant pathological consequences [11].

Proper recognition of the polypyrimidine tract (PPT) is essential for accurate 3′ splice site identification. Progressive deletions within the PPT have been shown to impair correct lariat formation, spliceosome assembly, and subsequent splicing. In addition, the characteristics of the PPT can modulate 3′ splice site selection by promoting the use of alternative branch sites [16,32,33].

Furthermore, there are several reports indicating that aberrant splicing can be associated with a missense or a silent change in an exon, rather than with mutations in introns. These findings suggest that such exonic mutations are capable of altering the secondary structure of the pre-mRNA, thereby interfering with the correct recognition of splicing regulatory elements [23,29,34].

## 3. *CSN1S1* and *CSN1S2* Gene Structure

Caseins account for about 80% of ruminant milk proteins. Moreover, they have a high nutritional value due to the contents in common and essential amino acids [35].

The αs-caseins are the most hydrophilic of all caseins. The primary distinction between the αs1-casein and αs2-casein families is predicated on their divergent amino acid sequences.

The encoding genes, *CSN1S1* and *CSN1S2*, are known to influence both the qualitative and quantitative properties of milk in major ruminant and non-ruminant species of zootechnical interest. These genes are annotated in nearly all species and are particularly well characterized in domestic ruminants (cattle, *Bos taurus*; river buffalo, *Bubalus bubalis*; sheep, *Ovis aries*; and goat, *Capra hircus*) and some camelids (dromedary, *Camelus dromedarius*; bactrian camel, *Camelus bactrianus*; llama, *Lama glama*). Both genes are located within a gene cluster spanning approximately 250 kb (kilobase), which also includes the β-casein and κ-casein genes (*CSN2* and *CSN3*, respectively). This cluster is located on chromosome 6 in *C. hircus*, *O. aries*, and *B. taurus*; on chromosome 7 in buffalo (*B. bubalis*) [36,37,38,39,40]; and on chromosome 2 in *C. dromedarius*, *C. bactrianus*, and *L. glama* [41], in which it spans a region of about 190 kb. An exception to this chromosomal organization is found in mammals such as donkeys (*Equus asinus*), horses (*Equus caballus*), rabbits (*Oryctolagus cuniculus*), and rodents (*Rodentia*), which possess an additional copy of the *CSN1S2* gene. This indicates a recent paralogous gene duplication event [4,41,42,43,44,45,46].

It has been hypothesized that the *CSN1S1*, *CSN2*, and *CSN1S2* genes evolved from a primordial gene, likely through both intra and intergenic duplications of an ancestral gene consisting of four exons [47,48,49,50]. In contrast to *CSN2*, the *CSN1S1* and *CSN1S2* genes have undergone extensive exon duplication, exon skipping, and exon conversion events. These processes have resulted in an extremely fragmented gene architecture characterized by numerous small exons within the coding region. Due to this close evolutionary relationship, the 5′ non-coding region, the domains encoding the ubiquitous multiple phosphorylation sites, and the 3′ non-coding region of the mRNAs of *CSN1S1* and *CSN1S2* exhibit high levels of sequence homology and are well-conserved [49,51,52,53,54,55,56].

*CSN1S1* and *CSN1S2* exhibit a conserved gene organization among ruminants, with some differences in intron size. These variations are primarily associated to artiodactyl retroposons (such as short interspersed elements—SINEs—and long interspersed elements—LINEs) located within their introns and regulatory regions [39,41,55]. In dairy ruminants, the *CSN1S1* gene spans approximately 16.7 kb and is composed of 19 exons and 18 introns. The exons show significant size variability, ranging from a minimum of 24 base pairs (bp) (exons 5, 6, 7, 8, 10, 13, and 16) to a maximum of 385 bp (exon 19). The first exon, a 53 bp sequence, is entirely non-coding. The leader peptide and the first two amino acids of the mature protein are encoded by exon 2 (63 bp). The translation stop codon TGA is created by coupling the final di-nucleotide TG of exon 17 with the first nucleotide A of exon 18 [55,56,57].

A similar structure also characterizes the *CSN1S1* gene in equids (*Equidae*) and camelids (*Camelidae*). In particular, in *E. caballus* and *E. asinus*, it has 19 exons, in *C. dromedarius* 20 exons, and in the other *Camelidae* 21 exons, ranging in size from 18 bp (exon 14) to 387 bp (exon 21) [9,41,46].

Similarly, in dairy ruminants, the *CSN1S2* gene is composed of 18 exons, which vary in size from 21 bp (exon 4) to 267 bp (exon 18). The first exon (44 bp) is entirely non-coding. The highly conserved signal peptide, a 15 amino acid sequence (MKFFIFTCLLAVALA), is encoded by exon 2 (63 bp). The mature protein consists of 207 amino acids in *B. taurus* and *B. bubalis*, and 208 in *O. aries* and *C. hircus*. The translation stop codon, TAA, is located at nucleotides 10 to 12 of exon 17. For both *CSN1S1* and *CSN1S2*, all splice junctions conform to the consensus GT-AG rule [39,45,51,54,55,56,57,58,59].

This gene has also recently been characterized in other minor dairy species. In *Camelidae*, the entire gene spans 17 exons, with sizes ranging from 24 bp (exons 4, 8, 11, and 13) to 280 bp (exon 17), and it encodes a mature peptide of 187 amino acids. Exon 1 (48 bp) is non-coding, as are the first 12 bp of exon 2. The signal peptide, a 15 amino acid sequence, initiates translation with an ATG codon at the 13th nucleotide and is fully encoded by the subsequent 45 nucleotides of exon 2. The mature peptide consists of 187 amino acids, is encoded by the last six nucleotides of exon 2, and continues up to the first 9 bp of exon 16. The translation stop codon, TAA, starts at nucleotides 10 of exon 16 [9,60].

As previously reported in the *E. asinus* genome, two different *CSN1S2* genes, called *CSN1S2*I and *CSN1S2*II, have been found. The first is constituted by 19 exons, and it encodes for αs2-CN-I, a protein of 221 amino acids, while the latter, consisting of 16 exons, is likely the result of a gene duplication event. In contrast to humans (*Homo sapiens*), where both *CSN1S2* paralogs are non-functional, the *E. asinus CSN1S2*II gene is considered functional. This functionality suggests that it produces an mRNA that is subsequently translated into an αs2-CN II protein. Similarly to *E. caballus*, both genes are closely associated and have been mapped to chromosome 3 [4,44,46].

## 4. Variants Involving Canonical Splice Sites at *CSN1S1* and *CSN1S2* Loci Affecting Pre-mRNA Splicing

Among the different protein fractions of milk, caseins αs1 and αs2 represent those that have an evolutionary conserved role and, therefore, are the most investigated. Given their functional importance, intricate structure, and remarkable variability, the genes encoding these proteins serve as powerful molecular models for evolutionary research. They also provide valuable insights into the genetic architecture of lesser-studied species and aid in elucidating the phylogenetic relationships among mammalian and domestic animal species [61].

### 4.1. Polymorphisms at CSN1S1 Locus

The αsl-CN family constitutes one of the most important milk casein fractions in dairy livestock species and consists of major and minor components [62,63]. Its encoding gene (*CSN1S1*) shows the highest level of polymorphism among those encoding the remaining milk proteins. The number of alleles identified at this locus has increased significantly over the years. To date, at least 23 variants of the *CSN1S1* gene have been identified in goats (*C. hircus*). These variants are associated with qualitative and quantitative differences in αs1-casein content and have been clustered into four distinct categories based on their expression levels: ‘strong’ (A, A1, A2, A3, A0, B0, B1, B2, B3, B4, C, H, L, and M: approximately 3.5 to 4.2 g/L per allele), ‘intermediate’ (E, I, D1: ~1.1 to 1.6 g/L per allele), ‘weak’ (D, F, and G: ~0.45 to 0.6 g/L per allele), and ‘null’ alleles (01, 02, and N: 0.0 g/L per allele or trace). Additionally, two new αs1-casein variants, a ‘null’ and an ‘F-like’ variant, have recently been detected in an indigenous Norwegian goat population [64,65,66].

In *B. taurus* the *CSN1S1* locus shows ten alleles (A, B, C, D, E, F, G, H, I, and J), which correspond to nine protein variants. For the G variant specifically, a considerable reduction in αs1-casein content has been reported, which consequently leads to lower milk coagulation times and reduced curd firmness. This highlights the direct link between this genetic variant, its effect on protein quantity, and the resulting technological properties of the milk [67,68,69,70].

The nomenclature for milk proteins is unified across the four main species of the genus *B.* considered in cattle milk protein studies: *B. taurus* (taurine cattle), *B. indicus* (zebu), *B. grunniens* (yak), and *B. javanicus* (banteng) [68,70].

In *O. aries*, nine variants of the *αs1-*casein have been identified, namely A, B, C, D, E, F, G, H, and I, although not all have been fully characterized. In particular, the *CSN1S1* D allele is associated with a lower casein content, a reduced casein index, a greater whey protein content, and poorer lactodynamographic parameters [54,71,72,73,74,75,76].

This locus is equally polymorphic in buffalo. To date, the known variants are A, B, B’, B’’, C, D, E, and F, among which B’ and B’’ are synonymous variants of B, while eight amino acid deletions characterize variants E and F compared with the others. In particular, the F variant corresponds to the previously named αs1-CN variant BRV [77,78].

The ancestral B variant is shared by both river and swamp buffalo. Alleles A, B’, B’’, C, E, and F have been found in river buffalo, whereas the D variant is exclusive to swamp buffalo [58,77,78,79].

*CSN1S1* gene polymorphism has also been extensively examined in camelids and equids. In *C. dromedarius*, at least four variants (αs1-caseins A, B, C, and D) have been identified [41,80,81,82,83,84], while only two variants have been reported in *L. glama* [85]. At this locus, six alleles (A, A *, B, C, D, and E) have been characterized in *E. caballus* [86]. In contrast, =*E. asinus* has been less investigated but is known to possess a variant associated with an apparently null content of this protein fraction [87].

### 4.2. Variants Involving Canonical Consensus Splicing Regulatory Sequences at CSN1S1 Locus

During livestock evolution, the *CSN1S1* gene acquired many sequence variations that lead to altered gene expression. To date, at least 13 alleles (Table 1) originating from mutations that affect consensus splicing regulatory sequences have been identified at this locus.

#### 4.2.1. *Bos taurus*

The *CSN1S1* A variant of *B. taurus* lacks 13 amino acids (^14^EVLNENLLRFFVA^26^) if compared with the B variant. This deletion stems from the corresponding absence of 39 bp (the entirety of exon 4) in the mRNA. This exon skipping event is directly correlated with a single point mutation (T > A transversion) at position +6 in the splice donor sequence distal to exon 4. This specific mutation impairs the normal splicing process of the A allele’s αs1-casein pre-mRNA, leading to the skipping of the upstream exon [77,88]. The authors postulate that the A allele-specific mutation at position +6 disrupts the perfect complementarity between the intron-4 splice donor signal (positions +1 to +8) and U1-snRNP, thus preventing the U1-snRNP from compensating for a rather weak upstream splice acceptor sequence of exon 4, which is crucial for facilitating the initial binding of the U2AF65 protein to the polypyrimidine tract. This mechanism effectively explains why the A variant’s mRNA lacks exon 4.

Furthermore, the analysis of the *CSN1S1* gene in a New Zealand herd producing A variant protein (A1) revealed another mutation, a single base deletion (Adenine) in position 4 of the 5′ splice sequence of the same intron. However, the net result is identical, namely, exon 4 skipping during mRNA processing. It was hypothesized that, due to the deletion of the 13 amino acid residues, the A variant lost a major chymosin cleavage site and had a less hydrophobic nature than its other counterparts in the αs1-casein family. Therefore, the physico-chemical properties may be different from other αs1-casein variants [89].

A similar molecular event was also presumed for a third allele at the bovine *CSN1S1* locus named H, which is characterized by the skipping of exon 8 encoding the ^51^NQAMENIK^58^ peptide [90]. Bovine *CSN1S1* A and H alleles are not common. Variant A was reported only in Holstein Friesian, Red Holstein, and German Red breeds with an allele frequency between 0.01 and 0.001, while *CSN1S1* H was present only in German Friesian (0.002) and African Kuri cattle (0.04) [90,97,98,99].

#### 4.2.2. *Ovis aries*

Similarly to the bovine species, the ovine *CSN1S1* locus shows three alleles characterized by mutations in canonical splicing sites *CSN1S1* E, H, and I.

The *CSN1S1* E variant lacks the amino acids 70 to 77 (EIVPNSAE), corresponding to the entirety of exon 10. This deletion also causes the loss of four phosphoserine (SerP) residues located at sites 64, 66, 68, and 75. The reduced content of αs1-casein E could therefore have a detrimental effect on the cheese-making properties of the milk and its mineral carrier activity. However, no further molecular genetic analyses are available [76,91,100].

As in the *B. taurus CSN1S1* H allele, the skipping of exon 8 also characterizes the *CSN1S1* H allele in the ovine species. It has been demonstrated that the deletion of four nucleotides (the last three of exon 8 and the first one of intron 8), replaced by a 13-nucleotide insertion in the DNA sequence, is the mutation underlying skipping of exon 8, leading to variant H production. This event inactivates the splice donor sequence distal to exon 8, leading to its skipping during the serial splicing reactions of the ovine αs1-casein pre-mRNA. Consequently, the mature protein lacks the peptide sequence ^51^DQAMEDAK^58^, which is normally encoded by exon 8 [72].

Finally, the sequencing of *CSN1S1* cDNA and the mature protein led to the identification and characterization of the *CSN1S1* I allele, carrying the constitutive skipping of exon 7. This allelic aberration is correlated with a sequence difference (T > A) in the 5′-splice donor sequence of intron 7, which disrupts the crucial base pairing with U1-snRNA. As a result, the mature mRNA sequence of ovine *CSN1S1* I is 24 bp shorter than the wild-type coding sequence. This deletion leads to the absence of eight amino acids (^43^DIGSESIE^50^) in the mature protein, including two phosphorylated serine residues (p.SerP46 and p.SerP48) [73]. Beyond the loss of these two phosphoserine residues, the deleted sequence also contains three acidic residues (one Asp and two Glu). These residues are critical for forming electrostatic bonds with calcium ions, which contributes to higher micellar size and improved curd consistency. Consequently, this variant has a reported negative impact on all milk technological properties, with a significant effect on protein yield and content [73,74].

Furthermore, it was observed that the *CSN1S1* I allele is expressed at the same level as the full-length variants, while the deleted ovine *CSN1S1* E and H alleles show a lower expression [73,91]. Allele frequencies of the E, H, and I αs1-CN variants are generally low and occur only in some breeds. αs1-CN H seems to be characteristic for East Friesian dairy sheep (allele frequency from 0.08 to 0.1869), whereas αs1-CN I (allele frequency 0.029) and E (0.003) were identified only in the Gray Horned Heath and Leccese breeds, respectively [72,73,74,101,102].

#### 4.2.3. *Capra hircus*

In *C. hircus*, two protein variants are known to originate from mutations at canonical splice sites at the *CSN1S1* locus. A deletion of 11 amino acids (^59^QMKAGSSSSSE^69^) characterizes the variant named *CSN1S1* D. This protein sequence is encoded by exon 9, which comprises the major phosphorylation site of the protein [103]. Splice site mutations are, according to some reports, the cause of this deletion; they would, in fact, cause the alteration of RNA processing and the exclusion of exon 9 in the mature RNA assembly [92,103].

Similarly, the protein variant αs1-CN G lacks 13 amino acids (^14^EVLNENLLRFVVA^26^) corresponding to exon 4. In this case, the skipping is caused by a G > A transition at the first nucleotide of the 5′ splice site consensus sequence at the beginning of intron 4 [104].

Both *CSN1S1* G and D alleles are associated with low αs1-casein content in milk [93,104]. Unfortunately, to date, there are few studies evaluating the frequency of these alleles in different goat breeds/genetic types. The only report is related to the *CSN1S1* D allele, the frequency of which in a large Alpine herd of Moissac was 0.025. It was also observed with a low frequency in the Saanen breed [92].

#### 4.2.4. *Bubalus bubalis*

In *B. bubalis*, to date, two alleles of the *CSN1S1 locus* characterized by alteration of a canonical splice site are known: variants E and F. The first may directly originate from variant A through constitutive exon 6 skipping (involving 24 nucleotides), while variant F could originate from the B allele, as it shares the same amino acid substitutions at positions 31 and 44 but exhibits the same skipping phenomenon as the E allele. Based on exon skipping alone, both alleles are commonly grouped into a single allele, called *CSN1S1* Bbt or BRV [78]. This exon skipping event triggers the synthesis of a defective protein lacking eight amino acids (EKVNELST) between positions 35 and 42 of the mature protein. The molecular weight of the deleted mutant is 919 Da less than the wild type. The molecular event responsible for exon 6 skipping during mRNA maturation in *CSN1S1* Bbt alleles has been identified as a G to C substitution at the first position of intron 6. This mutation inactivates the donor splice site, leading to the observed exon skipping [77,105].

Stepic et al. [106] observed that *CSN1S1* Bbt was associated with higher protein and casein content in milk, but its effect on milk composition and its technological properties requires further investigation.

The *CSN1S1* Bbt allele has a frequency of 0.18 in Romanian buffaloes [77] and 0.23 in a Serbian buffalo population [106]; a similar frequency is also thought to occur in animal populations from Venezuela, Bulgaria, Poland, and Canada. In contrast, the deleted αs1-casein variant did not occur in a Mediterranean Italian River Buffalo breed reared in Italy [105,107].

#### 4.2.5. *Camelus dromedarius*

The *CSN1S1* gene of *C. dromedarius* consists of 20 exons, unlike in other camelids, in which it is divided into 21 exons. The main difference arises from exon 20 (44 bp), which partially codes for the termination stop codon (exon 19 5′-TG … A-3′ exon 20). The cause of this difference lies in a mutation that occurred exclusively at the donor splice site of the dromedary sequence, which alters the correct identification of the splicing sites and results in the skipping of the exon. The correct reading frame is subsequently restored by the next exon, which also begins with an adenine, thereby re-establishing the termination stop codon. In other camelids, both exon 20 and the corresponding splicing sites are conserved [9,41]. Therefore, it should not be considered an intra-species variant, but rather a constitutive event of *C. dromedarius*.

In addition, the presence of genomic variants at the *CSN1S1* locus, originating from mutations in consensus splicing regulatory sequences, has been hypothesized for dromedary camels. In particular, three alleles, *CSN1S1* A, C and D, would appear to be characterized by the absence of exon 18 (exon 16 using bovine exon numbering) at the mRNA level (encoding for the peptide ^155^EQAYFHLE^162^) [80,81] compared with the *CSN1S1* B allele found by Kappeler et al. [108], with a consequent difference in the length of the corresponding protein (207 amino acids vs. 215). However, the sequence of exon 18 is present at the DNA level in all three alleles. Therefore, the protein variant could originate by the skipping out of the exon during mRNA processing, probably as a consequence of a not well characterized mutation at the DNA level altering the pre-mRNA spliceosome machinery. It is assumed that the mutational event underlying this variant is an 11 bp insertion (ATTGAATAAAA) located within intron 17, specifically positioned between the branch point sequence and the polypyrimidine tract immediately upstream of exon 18. The change in the αs1-CN peptide pattern due to the absence of this exon may modify the IgE-binding epitopes and alter the availability of bioactive peptides [81].

Furthermore, the *CSN1S1* C allele is characterized with respect to the remaining alleles at this locus by a non-synonymous single-nucleotide polymorphism G > T occurring at exon 5, leading to the amino acid substitution p.Glu30 > Asp in the mature protein and responsibility for Isoelectric Point change (3.22 vs. 2.77). On the other hand, no further information is reported for the D allele [80,81]. Therefore, it is to be considered that C and D are synonymous variants of the A.

The variant missing exon 18 seems to be the usual form within *C. dromedaries CSN1S1* mRNA and αs1-CN protein. In fact, the frequencies of the alleles without this exon have been found to be higher, with dominance of *CSN1S1* A (frequency ranging from 1.00 to 0.79) and a lower frequency of the C (0.00–0.28) and D (0.020–0.150) alleles [80,81,84].

### 4.3. Polymorphisms at CSN1S2 Locus

The *CSN1S2* locus was also found to be particularly polymorphic in the four main dairy ruminant species. To date, in *O. aries*, seven alleles have been characterized (*CSN1S2* A, B, C, D, E, F, and G) [109], while at least eight alleles have been identified to date in buffalo: *CSN1S2* A, B, B1, B2, C, D, E, and F [39,110].

The most remarkable polymorphism in this locus has been found in *C. hircus*. Currently, several alleles associated with different αs2-CN expression levels have been characterized. Eight alleles of the *CSN1S2* gene are known in *C. hircus*, and they are associated with three different quantities of αs2-CN in milk. These variants are clustered as follows: normal (*CSN1S2* A, B, C, E, F, and G), corresponding to ~2.5 g/L of αs2-casein per allele; intermediate (D), corresponding to ~1.5 g/L per allele); or null (0), resulting in no detectable amount of this casein [95,111,112,113,114]. Four new non-defective αs2-CN variants (H, I, J, and K) were detected in domestic breeds and wild goat species reared in Sudan [115] and eight novel variants (L, M, N, O, P, Q, R, and S) were found in Indian goats [116].

To date, the studies carried on *B. taurus* have identified only five variants (A, B, C, E, and D) [62,69]. Among these, the B and C alleles seem to be specific for the *B. indicus* and *B. grunniens*, respectively [117].

The high level of polymorphism at the *CSN1S2* locus is not considered unusual. Studies of the αs2-casein have revealed extensive polymorphisms at both the protein and DNA levels across various species. Indeed, the characterization of the *CSN1S2* gene in Old World camels and the *CSN1S2*I and II genes in *E. asinus* and *E. caballus* has also revealed interesting genetic variations. In horses, a total of six mutations leading to eight distinct putative protein isoforms (*CSN1S2*I A, B, C, D1, D2, E1, E2, F) were identified. In particular, the horse *CSN1S2*I B variant is a αs2-CN short form as it is characterized by the presence of a 1.3 kb in-frame deletion spanning two coding exons corresponding to 17 amino acid residues [86,118,119].

The *CSN1S2* I variant of *E. asinus* has seven SNPs at the mRNA level, five of which led to amino acid changes, while at the *CSN1S2*II locus, nine SNPs were observed, seven of which are non-synonymous [4,46].

Currently, the molecular mutations that cause most of the above-mentioned *CSN1S1* and *CSN1S2* alleles are known, from single-nucleotide substitutions/deletions to large insertions/deletions.

It has been observed that the presence of specific genomic variants affecting the consensus splicing regulatory sequences in these two genes can modify the splicing process. This leads to partial or complete exon loss or intron gain in the mature mRNA, which ultimately alters the corresponding protein-coding sequence.

### 4.4. Variants Involving Consensus Splicing Regulatory Sequences at CSN1S2 Locus

Although less frequently, polymorphisms that affect consensus splicing regulatory sequences are also documented at the *CSN1S2* locus in several livestock species of interest. This may be a consequence of the more limited number of studies conducted at this locus. In fact, to date, these variants have been described only in bovine, caprine, bubaline, asinine, and camelid species (Table 1).

#### 4.4.1. *Bos taurus*

The deletion of the nine amino acid residues, ^51^EYSIGSSSE^59^, characterize the αs2-CN D variant in *B. taurus*. The mutational event leading to the CSN1S2 D allele is the transversion c.221G > T, located at the last nucleotide of exon 8, corresponding to the 5′ consensus splicing site. This is considered the cause of the altered splicing of the primary transcript of αs2-CN D. Six taurine breeds (Angler, German Yellow, Hinterwälder, Limpurger, Hungarian Grey Steppe, and Vorderwälder) carry this allele, albeit at low frequency (ranging from 0.02 to 0.10), while it was not reported in indicine breeds [69,94].

#### 4.4.2. *Capra hircus*

The *CSN1S2* D allele shows a 106 bp deletion, from the last 11 bp of exon 11 and the first 95 bp of the following intron. As a result of this deletion, exon 11 loses the canonical donor splice site. Moreover, the last undeleted nucleotide (A) of exon 11 (the first of codon 121) and the two nucleotides of the following intron (AT) could form a new codon corresponding to Asn121, followed by a new GT dinucleotide splicing donor site with lacking at least three codons coding for Pro122, Thr123, and Val124. This variant is 205 vs. 208 amino acids long [113]. However, a preliminary analysis of the transcripts produced by the *CSN1S2* D allele was unable to detect mRNA characterized by this event. Conversely, transcripts characterized by the skipping of the entirety of exon 11 (123 nt, coding for the peptide ^84^NEINQFYQKFPQYLQYPYQGPIVLNPWDQVKRNAGPFTPTV^124^) have been observed, which would consequently translate into a protein of only 167 amino acids [96].

Like most defective alleles arising from mutations affecting canonical splice sites, the goat *CSN1S2* D, apparently associated with decreased synthesis of αs2-CN in goat milk, is a rare allele. To date, its presence has been demonstrated in the “Argentata dell’Etna” (0.012) and “Girgentana” (0.006) breeds, as well as in an undefined genetic type belonging to a local population (frequency 0.019), all reared in southern Italy. It has also been found in Saanen (0.174) and Thai Native (0.143) goats raised in Thailand, and in Hungarian goats (0.005) [113,120,121,122]. In contrast, the allele appears to be absent in autochthonous goat breeds reared in Northern and Central Italy [66,123,124,125,126], as well as in populations from Turkey [127,128] and the Czech Republic [129].

#### 4.4.3. *Bubalus bubalis*

The deleted allele *CSN1S2* B identified in *B. bubalis* carries an SNP (FM865620:g.773G > C) positioned at the donor splice site of exon 7 (27 bp). This mutation causes the complete skipping of exon 7 and the deletion of nine amino acids (^42^EVIRNANEE^50^) in mature protein. Consequently, the predicted mature bubaline αs2-CN B protein is 198 aa long instead of 207 aa [110]. More recently, two other B-derived alleles have been described, named *CSN1S2* B1 and B2, characterized by the same mutation at the intron 7 splice donor site but differing for single polymorphisms (haplotypes), with B2 probably arising from interallelic recombination (single crossing) between the alleles D and B (or B1) [39]. An association study between the SNP g.773G > C and milk traits, including fatty acid composition, was carried out, but no significant associations were observed [39,130].

To date, the frequency of this mutation has been estimated only in Italian Mediterranean buffalo reared in Campania and Basilicata regions (Southern Italy), and it was found to be 0.17 [39,110].

#### 4.4.4. *Equus asinus*

Mutation of splice junctions sometimes leads to activation of cryptic splice sites. In fact, it has been demonstrated that *E. asinus* carries a characteristic polymorphism (FM946022.1: c.375-1G > A) at the splice acceptor site of *CSN1S2*I exon 17. This point mutation results in allele-specific skipping of the first 15 nucleotides of this exon, which encode the peptide ^176^NKINQ^180^. Concurrently an in-frame cryptic splicing acceptor site (AACAAAATCAACCAG) is recognized. While the presence of the adenine, responsible for skipping the first 15 nucleotides of exon 17, is observed at a low frequency in *E. asinus* (0.2437), it appears to be a constitutive condition in all other species across different families within the *Perissodactyla* order. All these other *Perissodactyla* species are characterized by the presence of a second in-frame splice acceptor site, which enables recognition of the exon 17 deletion of the first 15 nucleotides during the splicing mechanism. Therefore, this case represents an intra-species allele-specific event, rather than a constitutive condition, as reported for *E. caballus*. In fact, it has been suggested that the G allele represents the ancestral condition of the *CSN1S2* gene. During the evolution of *Perissodactyla*, a G > A transition may have occurred, resulting in the fixation of adenine in all families of this order, except in *E. asinus* [4]. A similar evolutionary pattern was previously described in camelids at the same locus.

The frequency of adenine at the splice acceptor site of *CSN1S2*I exon 17 was found to be 0.2437 in Ragusana and Amiatina donkeys reared in different Italian regions [4].

#### 4.4.5. *Camelus dromedarius*

Recently, also in *C. dromedarius*, a mutation affecting splice site at the *CSN1S2* locus has been identified: c.403-9_403-4delTTTTCT. The splice site variant, which has a frequency of 0.323 in Tunisian dromedaries, is hypothesized to have a strong influence on protein function and stability. However, limited information has been reported regarding the specific functional consequences of this event [60,83].

## 5. Discussion and Future Perspectives

This review provides a comprehensive look at mutations affecting consensus splicing regulatory sequences in the *CSN1S1* and *CSN1S2* genes of dairy livestock species. The prevalent finding is that most mutations leading to constitutive exon skipping at these loci primarily impact the 5′ donor splice sites. This pattern is consistent with human studies, in which point mutations at the 5′ donor splice site are more common than those at the 3′ acceptor site (62% vs. 26%). The functional consequence of mutations at donor sites is thought to be a significant reduction in the pairing between the splice site and the complementary sequence in the U1 small nuclear ribonucleoprotein particle (U1snRNP), a critical early step in the intricate process of mRNA splicing [131].

These alterations in pre-mRNA splicing patterns add to the already widely investigated effects of alternative splicing in various species. In goats, for example, specific polymorphisms can introduce premature termination codons (PTCs), leading to the accumulation of a minor alternative splice form where an exon is often skipped. This can trigger nonsense-mediated decay (NMD), profoundly impacting the expression levels of the encoded αs1 and αs2 caseins [7,95,132].

A growing number of health benefits and a diverse range of potential activities are attributed to milk proteins and the bioactive peptides encoded within their sequences. Caseins, particularly αs1-CN and αs2-CN, are known to be significant sources of these peptides [15,133]. Consequently, it is plausible that the truncated proteins resulting from constitutive exon skipping—a direct consequence of mutations in splice sites—could lead to the emergence of new allergenic epitopes or the alteration of existing IgE-binding epitopes. This would, in turn, affect the availability of valuable bioactive peptides. This hypothesis is supported by similar proposals for various protein isoforms with rearranged amino acid sequences that arise from normal alternative splicing events characteristic of casein-coding loci in different dairy livestock species [134]. Likewise, allele-dependent alternative splicing events could lead to similar outcomes. For instance, skipping of exon 18 of the A, C, and D alleles at the *CSN1S1* locus in the *C. dromedarius*, or of the first 15 nucleotides of exon 17 encoding the pentapeptide ^176^NKINQ^180^ in the *E. asinus* αs2-CN variant, is hypothesized to change the casein peptide pattern and affect IgE-binding epitopes, thereby altering the availability of bioactive peptides [4,81].

In general, alternative splicing results in protein isoforms that typically characterize the protein pool from a single gene to a lesser extent, regardless of genetic variability. Conversely, with allele-specific splicing, the altered protein produced represents the primary translated product and possesses a unique amino acid sequence. Because this is a direct consequence of a precise mutational event, it becomes possible to conduct DNA-level selection for or against a specific variant to improve the functional quality of milk and its derived products. The loss of an entire exon or a portion of it can drastically alter the protein’s three-dimensional structure, stability, and post-translational modifications (e.g., phosphorylation sites), leading to a cascade of effects on its biological function.

Future research should focus on a multidisciplinary approach to fully understand the implications of these findings. Identifying novel allele-specific splicing events across diverse dairy livestock species is a critical first step. Advanced omics technologies will be instrumental in this endeavor. Long-read transcriptomics (e.g., PacBio, Oxford Nanopore) is particularly valuable as it allows for the sequencing of full-length transcripts without the need for assembly, providing an accurate and complete profile of all splice isoforms from a single allele. Allele-resolved RNA sequencing will be crucial for distinguishing between the transcripts produced by different alleles within a heterozygous individual, offering an unprecedented level of detail on the regulatory mechanisms at these loci.

Translating genetic findings into practical innovations requires a deeper understanding of the functional consequences. Future studies should integrate proteomics, using techniques like mass spectrometry, to confirm that the identified mRNA isoforms are translated into functional proteins and to characterize their precise amino acid sequences and post-translational modifications. Furthermore, in vitro and in vivo functional studies will be essential to evaluate the nutritional, allergenic, and technological implications of these modified proteins. For example, cellular models could be used to test the immunogenicity of milk peptides from different alleles, while animal studies could assess the allergenic potential of milk from specific genotypic variants.

A deeper understanding of allele-specific splicing may enable precision breeding strategies aimed at producing milk with improved functional properties, reduced allergenicity, or enhanced technological performance for cheesemaking. The ability to select animals based on specific genotypic markers linked to beneficial splicing outcomes offers a powerful tool for modern livestock management. Such advances could support the development of differentiated dairy products tailored to specific consumer needs, such as low-allergenicity milk or milk with a higher content of specific bioactive peptides. This approach not only adds value to the dairy supply chain but also contributes to more sustainable and economically viable production systems.

## Figures and Tables

**Table 1:** Alleles by alteration of consensus splicing regulatory sequences at *CSN1S1* and *CSN1S2* loci.

*Locus*	Species (Common Name)	Alleles	Constitutive Exon Skipped	PEPTIDE Deleted *	Involved Consensus Splicing Sequences	References
*CSN1S1*	*Bos taurus* (Cattle)	A; A1 H	Exon 4 Exon 8	^14^EVLNENLLRFFVA^26^ ^51^NQAMENIK^58^	Donor splice sites	[88,89,90]
	*Ovis aries* (Sheep)	H I E	Exon 8 Exon 7 Exon 10	^51^DQAMEDAK^58^ ^43^DIGSESIE^50^ ^70^EIVPNSAE^77^	Donor splice sites	[72,73,91]
	*Bubalus bubalis* (Water buffalo)	E (Bbt) F (Bbt)	Exon 6	^35^EKVNELST^42^	Donor splice site	[77,78]
	*Capra hircus* (Goat)	D G	Exon 9 Exon 4	^59^QMKAGSSSSSE^69^ ^14^EVLNENLLRFVVA^26^	Donor splice sites	[92,93]
	*Camelus dromedarius* (Dromedary)	A; C; D	Exon 18	^155^EQAYFHLE^162^	Branch sequence and polypyrimidine tract	[80,81]
*CSN1S2*	*Bos taurus* (Cattle)	D	Exon 8	^51^EYSIGSSSE^59^	Donor splice site	[94]
	*Bubalus bubalis* (Water buffalo)	B; B1; B2	Exon 7	^42^EVIRNANEE^50^	Donor splice site	[39]
	*Capra hircus* (Goat)	D	Exon 11	^84^NEINQFYQKFPQYLQYPYQGPIVLNPWDQVKRNAGPFTPTV^124^	Donor splice site	[95,96]
	*Equus asinus* (Donkey)	Unnamed	First 15 nucleotides of exon 17	^176^NKINQ^180^	Acceptor splice site	[4]
	*Camelus dromedarius* (Dromedary)	Unnamed	Uncharacterized	Uncharacterized		[83]

* aa number relative to mature proteins.

## Abbreviations

The following abbreviations are used in this manuscript:

pre-mRNA	precursor messenger RNA
snRNPs	small nuclear ribonucleoprotein particles
SINEs	short interspersed elements
LINEs	long interspersed elements
bp	base pairs
PTCs	premature termination codons
